# Supplementary material for: Mutant fixation in the presence of a natural enemy
Source: Nat Commun. 2023 Oct 20;14:6642. doi: 10.1038/s41467-023-41787-5 (PMC10589345; doi:10.1038/s41467-023-41787-5)
Supplement: Supplementary file 1 — Supplementary Information [file 41467_2023_41787_MOESM1_ESM.pdf]

Mutant fixation in the presence of a natural enemy

Supplementary Information

1 Supplementary Note 1: A coarse-grained approach in the absence of mutants

1.1 The patch model of infected and uninfected cells and stochastic simulations

Consider the patch model described in the main text. We first focus on the basic dynamics in the absence of mutants. There are $\mathcal{N}$ patches, and cells migrate between patches randomly and uniformly, regardless of their location. Denote by x_i and z_i the populations of uninfected and infected cells in deme i, respectively. The co-dynamics of cells and infection within patches happen according the following ODEs:

$$\dot{x}_i = rx_i\left(1-\frac{x_i+z_i}{K}\right) - dx_i - \beta x_i z_i + \mu\left(\frac{\sum_{k=1}^{\mathcal{N}} x_k}{\mathcal{N}} - x_i\right), \quad (1)$$

$$\dot{z}_i = \beta x_i z_i - a z_i + \mu\left(\frac{\sum_{k=1}^{\mathcal{N}} z_k}{\mathcal{N}} - z_i\right), \quad 1 \le i \le \mathcal{N}, \quad (2)$$

where r and d are respectively the linear growth and death rates of uninfected cells, K is the demes' carrying capacity, β is the infection rate, a is the death rate of infected cells, and μ is the migration rate of cells. Equations (1-2) describe the version of the model characterized by non-spatial migration, that is, migration happens among all the $\mathcal{N}$ patches. To study the nearest-neighbor migration, we would replace the term containing μ in equation (1) with

$$\sum_{k=1}^{\mathcal{N}} \mu_{ik}(x_k - x_i),$$

and similarly, for equation (2), where $\{\mu_{ik}\}$ is a symmetric migration matrix. To implement migration to the nearest neighbors, we placed the patches on a square grid with periodic boundary conditions, and set $\mu_{ij} = \mu/8$ if patches i and j are neighbors in the Moore neighborhood sense, and $\mu_{ij} = 0$ otherwise.

Here we will describe the Gillespie-type stochastic simulation algorithm that we used to sample trajectories from the mean-field description, by using the example of system (1-2). Our algorithm is one of many possible stochastic methodologies, see e.g. [1] for a review. For convenience, in the stochastic system, let us suppose that the current number of uninfected individuals in patch i is denoted by x_i, and the current number of infected individuals in patch i is denoted by z_i (these are integer numbers in the stochastic simulation). This way, the various terms in equations (1-2) describe the propensities of all the possible stochastic events. For example, $\mathcal{P}_1^{(i)} = dx_i$ is the propensity for a death event of an uninfected individual in patch i, and $\mathcal{P}_2^{(i)} = \beta x_i z_i$ is the propensity for an infection event in patch i where a single individual becomes infected. For patch i, there are also the following propensities:

$$\mathcal{P}_3^{(i)} = \left[r x_i \left(1 - \frac{x_i + z_i}{K} \right) \right]_+, \quad \mathcal{P}_4^{(i)} = a z_i, \quad \mathcal{P}_5^{(i)} = \mu x_i, \quad \mathcal{P}_6^{(i)} = \mu z_i,$$

where $[\ldots]_+$ stands for the positive part. Let us define the total patch propensity $\mathcal{P}^{(i)} = \sum_{k=1}^{6} \mathcal{P}_k^{(i)}$ for $1 \le i \le \mathcal{N}$, and the total system propensity as $\mathcal{P} = \sum_{i=1}^{\mathcal{N}} \mathcal{P}^{(i)}$.

The algorithm proceeds as a sequence of steps. For each step, we first determine which patch is chosen for an updating event. This is done by choosing patch i with probability $\mathcal{P}^{(i)}/\mathcal{P}$ for each i. Once the updating patch is chosen, we need to determine what update happens. This is done by picking update k with probability $\mathcal{P}_k^{(i)}/\mathcal{P}^{(i)}$ for each $k \in \{1, \ldots, 6\}$. Once the update is chosen, the number of individuals is changed accordingly. For example, if update $k = 2$ is chosen, then the number of individuals in patch i changes as $x_i \to x_i - 1$ and $z_i \to z_i + 1$. Note that if a migration update is chosen (for example, $k = 5$), then the number of individuals in patch i decreases ($x_i \to x_i - 1$), and also the number of individuals increases in a randomly chosen patch m (with all patches being equally likely to be picked): $x_m \to x_m + 1$. Finally, we determine the time-increment, that is, the length of time, Δt, that elapsed between the previous update and the current update. This is done by setting Δt to be a random number exponentially distributed with the mean $1/\mathcal{P}$. Note that our algorithm follows the description of Appendix A, Direct Method, in Ref. [1].

Trajectories obtained by using this algorithm are characterized by the mean values approximately given by equations (1-2).

To numerically study mutant fixation in the patch model, a procedure similar to the ABM was followed. Initially, a square block of patches was populated with a number of wild-type, uninfected individuals, and in the middle of that block, a smaller block of patches contained both infected and uninfected, wild-type individuals (the precise numbers used were 30 uninfected individuals per patch, and in addition 10 infected individuals per patch in the infected block). The stochastic algorithm described above was followed until a quasi-equilibrium state was reached, at which point mutant(s) were introduced, as described in the main text.

1.2 A coarse-grained description

Let us define an infected patch as a patch that contains at least one infected cell (and it may or may not contain uninfected cells). An uninfected patch contains at least one uninfected cell and no infected cells. Instead of focusing on the dynamics of infected and uninfected cells within patches, we can consider the populations of infected and uninfected patches within the grid, see Supplementary Figure 1.

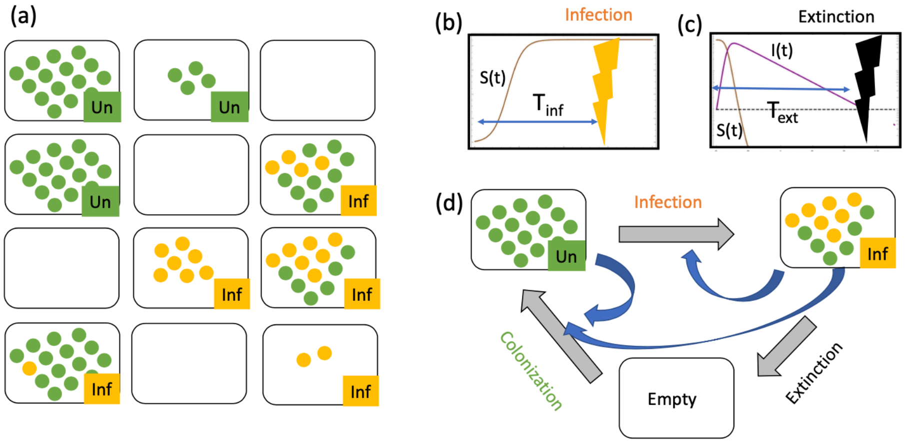

Supplementary Figure 1: A schematic of the coarse-grained model. (a) Patches can be uninfected, infected, or empty; here green and orange dots denote uninfected and infected cells. (b) An infected patch can become infected after a time, T_{inf}. (c) An infected patch can become extinct after a time, T_{ext}. (d) The coarse-grained description includes these two processes and a process of colonization of empty patches. Here the blue arrows indicate which populations contribute to which rates.

Suppose the number of uninfected patches is X, and the number of infected patches is Z. Let us denote the mean number of cells in an uninfected patch as $w_x^{(X)}$; the mean number of infected cells in an infected patch as $w_z^{(Z)}$, and the mean number of uninfected cells in an infected patch as $w_x^{(Z)}$. We have the following coarse-grained dynamics of patches:

$$\dot{X} = \mu(Xw_x^{(X)} + Zw_x^{(Z)})\left(1 - \frac{X+Z}{\mathcal{N}}\right)P_{col} - \mu Z w_z^{(Z)}\frac{X}{\mathcal{N}}P_{inf}, \quad (3)$$

$$\dot{Z} = \mu Z w_z^{(Z)}\frac{X}{\mathcal{N}}P_{inf} - \frac{Z}{T_{ext}}. \quad (4)$$

Here, the first term in the right hand side of equation (3) is the rate at which all the uninfected cells in the system $(Xw_x^{(X)} + Zw_x^{(Z)})$ migrate and land on an empty patch (probability to hit an empty patch is $1-(X+Z)/\mathcal{N}$), which is followed by a successful colonization (probability P_{col}). The second term in equation (3), as well as the first term in equation (4), describes the rate at which infected cells $(Xw_z^{(Z)})$ migrate to one of the uninfected patches (probability $X/\mathcal{N}$), such that takes off (probability P_{inf}). The last term in equation (4) is the rate at which infected patches go extinct, with T_{ext} denoting the mean extinction time of an infected patch. The probability of successful colonization, P_{col}, and the probability of successful infection spread, P_{inf}, can be approximated as follows,

$$P_{col} = 1 - d/r, \quad (5)$$

$$P_{inf} = 1 - 1/R_0 = 1 - (w_x^{(X)}\beta/a)^{-1}. \quad (6)$$

Other parameters of system (3-4), such as the mean time of extinction, P_{ext}, and mean population sizes $w_x^{(X)}, w_z^{(Z)}$, and $w_x^{(Z)}$, are calculated from microscopic patch dynamics, as described next.

We will denote by $S(t)$ the number of uninfected cells and by $I(t)$ the number of infected cells in a typical patch.

Uninfected patches. We have, inside the uninfected patches:

$$\dot{S} = rS(1-S/K) - dS - \mu S + \mu(Xw_x^{(X)} + Zw_x^{(Z)})\frac{1}{\mathcal{N}}, \quad (7)$$

$$S(0) = 1. \quad (8)$$

The life-span of an uninfected patch is defined by the timing of infection, T_{inf}. Infections happen at the rate $\frac{1}{\mathcal{N}}\mu Z w_z^{(Z)} P_{inf}$, so we have

$$T_{inf} = \frac{\mathcal{N}}{\mu Z w_z^{(Z)} P_{inf}}. \tag{9}$$

The mean population of an uninfected patch is therefore

$$w_x^{(X)} = \frac{1}{T_{inf}} \int_0^{T_{inf}} S(t)\, dt. \tag{10}$$

Infected patches. We have, inside the infected patches:

$$\begin{aligned}
\dot{S} &= rS(1-(S+I)/K) - dS - \mu S + \mu(X w_x^{(X)} + Z w_x^{(Z)})\frac{1}{\mathcal{N}} - \beta S I, \\
\dot{I} &= \beta S I - aI - \mu I + \mu Z w_z^{(Z)} \frac{1}{\mathcal{N}}, \\
S(0) &= w_x^{(X)}, & (11)\\
I(0) &= 1. & (12)
\end{aligned}$$

The time to extinction, $T_{ext} > 0$, is approximated by obtaining the time that corresponds to the first minimum of the function $I(t)$. The mean population sizes are then given by

$$w_z^{(Z)} = \frac{1}{T_{ext}} \int_0^{T_{ext}} I(t)\, dt, \tag{13}$$

$$w_x^{(Z)} = \frac{1}{T_{ext}} \int_0^{T_{ext}} S(t)\, dt. \tag{14}$$

Coarse-grained method numerical procedure. The goal is, given all the system parameters, to predict the expected (infected and uninfected) patch numbers and the expected number of (infected and uninfected) individuals inside patches. Solutions (X, Z) of system (3-4), as well as values $w_x^{(X)}, w_z^{(Z)}$, and $w_x^{(Z)}$ were used to approximate these values. The following steps were implemented:

0) Use an initial guess for the 5 quantities, $(X, Z, w_x^{(X)}, w_z^{(Z)}, w_x^{(Z)})$.

1) Evaluate parameters P_{inf} and T_{inf}, equations (6) and (9).

2) Numerically solve the system for infected patches (equations (11-12)) up to some large value of time, T_{max}.

3) Obtain $T_{ext} > 0$ by locating the first minimum of the function $I(t)$ in $(0, T_{max})$.

4) Numerically solve the system for uninfected patches (equations (7-8)) up to the value T_{ext}.

5) Evaluate the updated values of $w_x^{(X)}$ using the solution in 4), equation (10); evaluate the updated values of $w_z^{(Z)}$ and $w_x^{(Z)}$ using the solution in 2), equations (13-14).

6) Perform an iteration of a discretized version of equations (3-4) to find updated values of X, Z:

$$\dot{X}^{new} = X^{old} + \Delta t \left(\mu(X^{old} w_x^{(X)} + Z^{old} w_x^{(Z)}) \left(1 - \frac{X^{old} + Z}{\mathcal{N}}\right) P_{col} - \mu Z^{old} w_z^{(Z)} \frac{X^{old}}{\mathcal{N}} P_{inf} \right), \quad (15)$$

$$\dot{Z}^{new} = Z^{old} + \Delta t \left(\mu Z^{old} w_z^{(Z)} \frac{X^{old}}{\mathcal{N}} P_{inf} - \frac{Z^{old}}{T_{ext}} \right). \quad (16)$$

7) Repeat steps (0-6), until convergence is reached.

1.3 Stochastic simulations and coarse-grained predictions

Stochastic Gillespie-type simulations of system (1-2) were performed, as described in Section 1.1. The simulations were initiated by placing a relatively small block of occupied patches in the middle of the grid, with the rest of the patches initially empty. All the occupied patches were uninfected except for a smaller block in the middle. For example, in a 40×40 grid, we started with 7×7 occupied patches in the middle, of which the central 3×3 block was infected. The initial populations of the occupied patches were just below the carrying capacity, and the initial population of infected patches contained a third of infected cells. Simulations of this type were run for a range of

migration rate values, μ. Supplementary Figure 2 shows typical dynamics inside a single patch in a multi-patch simulation, under a lower (panel (a)) and a higher (panel (b)) migration rate.

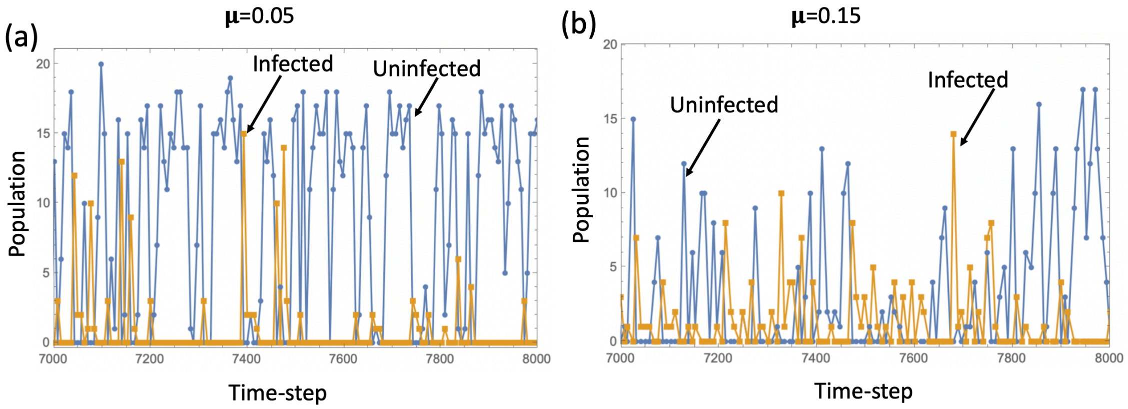

Supplementary Figure 2: Typical patch trajectories under (a) low migration ($\mu = 0.05$) and (b) high migration ($\mu = 0.15$). The numbers of uninfected (blue) and infected (yellow) individuals are plotted as a function of time for a single patch. The rest of the parameters are: $r = 0.6, d = 0.1, \beta = 1.5, a = 0.5, K = 20, \mathcal{N} = 400$.

For each value of the migration rate, we obtained numerical counts of infected and uninfected patch numbers, as well as the numbers of individuals inside patches, at quasi-equilibrium. To count patch numbers, we assumed that a patch is uninfected if it contains at lest one uninfected individual and no infected individuals; a patch is infected if it contains at least one infected individual. Supplementary Figure 3 shows a comparison of stochastic simulations (dots with vertical bars, representing ensemble means and standard deviations) with the coarse-grained method predictions (dashed lines), for patch numbers (X, Z, see panel (a)) and the numbers of individuals ($w_x^{(X)}, w_z^{(Z)}, w_x^{(Z)}$, see panel (b)).

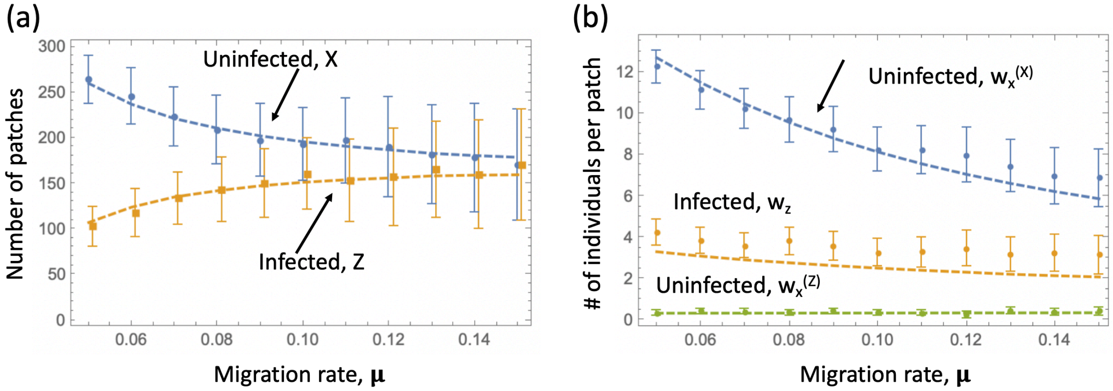

Supplementary Figure 3: Comparison of stochastic simulations (dots with vertical bars, showing means and standard deviations), and the coarse-grained method predictions (dashed lines). (a) The numbers of uninfected (blue) and infected (yellow) patches are plotted for different values of the migration rate, μ. (b) The numbers of uninfected individuals in uninfected patches ($w_x^{(X)}$, blue), infected individuals ($w_z^{(Z)}$, yellow), and uninfected individuals in infected patches ($w_x^{(Z)}$, green) are plotted for different values of μ. The rest of the parameters are as in Supplementary Figure 2.

2 Supplementary Note 2: A coarse-grained approach in the presence of mutants

2.1 The patch model in the presence of two types of hosts

In the presence of two types of hosts, the dynamics in the patch system are described by the following ODEs,

$$\dot{x}_i = r_x x_i \left(1 - \frac{x_i + y_i + z_i}{K}\right) - d_x x_i - \beta x_i z_i + \mu \left(\frac{\sum_{k=1}^{\mathcal{N}} x_k}{\mathcal{N}} - x_i\right), \quad (17)$$

$$\dot{y}_i = r_y y_i \left(1 - \frac{x_i + y_i + z_i}{K}\right) - d_y y_i - \beta y_i z_i + \mu \left(\frac{\sum_{k=1}^{\mathcal{N}} y_k}{\mathcal{N}} - y_i\right), \quad (18)$$

$$\dot{z}_i = \beta(x_i + y_i) z_i - a z_i + \mu \left(\frac{\sum_{k=1}^{\mathcal{N}} z_k}{\mathcal{N}} - z_i\right), \quad 1 \le i \le \mathcal{N}, \quad (19)$$

where x_i, y_i, and z_i are respectively the number of uninfected wild-type cells, uninfected mutant cells, and infected cells. The division rates of wild-type and mutant cells are given by r_x and r_y respectively. The death rates of wild-type and mutant cells are given by d_x and d_y respectively. It is assumed that while mutants may differ from the wild type cells by their division or death rates, both types of host are equally susceptible to infection (they are characterized by the same infectivity, β), they migrate with the same rate μ, and the death rate of infected cells, a, does not depend on their type. This is a generalization of system (1-2), and was also simulated by using the Gillespie-type algorithm.

2.2 A coarse-grained system in the presence of two types of hosts

Let us extend the coarse-grained approach, equations (3-4), to systems with two types of host, wild type and mutant cells. In order for this approach to work, most nonempty patches in the system should be purely wild-type or purely mutant. If a significant fraction of patches contains both types, the approach is not valid (see below for the applicability conditions). Denote by

X and Y the numbers of uninfected wild-type and mutant patches, respectively. The numbers of wild-type and mutant infected patches are denoted respectively by Z_x and Z_y. The coarse-grained system of equations in this case in given by

$$\begin{aligned}
\dot{X} &= -\mu(Yw_y^{(Y)} + Z_y w_y^{(Z_y)})\frac{X}{\mathcal{N}}P_{x\to y} + \mu(Xw_x^{(X)} + Z_x w_x^{(Z_x)})\frac{Y}{\mathcal{N}}P_{y\to x} \\
&+ \mu(Xw_x^{(X)} + Z_x w_x^{(Z_x)})\left(1 - \frac{X+Y+Z_x+Z_y}{\mathcal{N}}\right)P^x_{col} - \mu(Z_x w_z^{(Z_x)} + Z_y w_z^{(Z_y)})\frac{X}{\mathcal{N}}P^x_{inf}, \quad (20)\\
\dot{Y} &= \mu(Yw_y^{(Y)} + Z_y w_y^{(Z_y)})\frac{X}{\mathcal{N}}P_{x\to y} - \mu(Xw_x^{(X)} + Z_x w_x^{(Z_x)})\frac{Y}{\mathcal{N}}P_{y\to x} \\
&+ \mu(Yw_y^{(Y)} + Z_y w_y^{(Z_y)})\left(1 - \frac{X+Y+Z_x+Z_y}{\mathcal{N}}\right)P^y_{col} - \mu(Z_x w_z^{(Z_x)} + Z_y w_z^{(Z_y)})\frac{Y}{\mathcal{N}}P^y_{inf}, \quad (21)\\
\dot{Z}_x &= \mu(Z_x w_z^{(Z_x)} + Z_y w_z^{(Z_y)})\frac{X}{\mathcal{N}}P^x_{inf} - \frac{Z_x}{T^x_{ext}}, \quad (22)\\
\dot{Z}_y &= \mu(Z_x w_z^{(Z_x)} + Z_y w_z^{(Z_y)})\frac{Y}{\mathcal{N}}P^y_{inf} - \frac{Z_y}{T^y_{ext}}. \quad (23)
\end{aligned}$$

As before, the populations of individuals in different patches are denoted with w, where the subscript refers to the population type (x/y for uninfected w.t./mutants, and z for infected cells), and the superscript refers to the patch type. In the presence of two types of hosts, some of the rates now also have a superscript that specifies the type of a patch where the process takes place. For example, P^x_{col} and P^y_{col} denote the probability of successful colonization of an empty patch by wild type and mutant cells, respectively, and are given by $P^x_{col} = 1 - d_x/r_x$, $P^y_{col} = 1 - d_y/r_y$.

Compared to system (3-4), the equations in the presence of mutants have two new terms. The first term in the right hand side of equations (20) and (21) describes the rate at which uninfected mutant cells (totaling $Yw_y^{(Y)} + Z_y w_y^{(Z_y)}$) migrate to an uninfected wild-type patch (probability $X/\mathcal{N}$), and subsequently, the patch is "converted" to becoming a mutant patch by means of a stochastic fixation of mutants (probability $P_{x\to y}$). Similarly, the second term in the right hand side of equations (20) and (21) describes the conversion of mutant patches to wild-type patches. The probability of fixation of mutants in a patch of wild-type individuals, and also that for wild-type individuals in a patch of mutants, are given by

$$P_{x\to y} = \frac{1 - 1/(1+s)}{1 - 1/(1+s)^{w_x^{(X)}}}, \quad P_{y\to x} = \frac{1-(1+s)}{1-(1+s)^{w_y^{(Y)}}}, \quad s = \frac{r_y d_x}{d_y r_x} - 1.$$

Here, s is the selection coefficient and $w_x^{(X)}$ and $w_y^{(Y)}$ are patch sizes for the two types of cells. The above formulas are the Moran process approximations for the fixation probabilities for populations with demographic fluctuations [2].

2.3 Quantification of selection dynamics

The total number of uninfected wild-type cells in system (20-23) is given by $Xw_x^{(X)} + Z_x w_x^{(Z_x)}$, and the total number of uninfected mutants by $Yw_y^{(Y)} + Z_y w_y^{(Z_y)}$. The two contributions are unequal, with the large majority of uninfected cells coming from uninfected patches. Therefore, we can ignore[1] the terms $Z_x w_x^{(Z_x)}, Z_y w_y^{(Z_y)}$ compared to $Xw_x^{(Z_x)}, Yw_y^{(Z_y)}$. The resulting 3-equation system is presented in the main text, where we denoted $Z = Z_x + Z_y$.

We would like to determine the selection pressure experienced by the mutant patches, given that they are at low numbers compared to the number of wild-type patches. To proceed, we will find the equilibrium solution of system (20-23) in the absence of mutants ($X = X_{eq}, Y = 0, Z_x = Z_{eq}, Z_y = 0$), and then linearize the system around this solution, to obtain the equation for $\dot{Y}$.

We have the following equilibrium solution in the absence of mutants:

$$X_{eq} = \frac{\mathcal{N}}{\mu P^x_{inf} T_{ext} w_z^{(Z)}}, \quad Z_{eq} = \frac{\mathcal{N} w_x^{(X)} P^x_{col}}{\mu w_z^{(Z)} P^x_{inf} T_{ext}} \frac{\mu w_z^{(Z)} P^x_{inf} T_{ext} - 1}{w_x^{(X)} P^x_{col} + w_z^{(Z)} P^x_{inf}}.$$

Linearizing equation (21), we obtain

$$\dot{Y} = (\nu_{col} + \nu_{x\to y} - \nu_{inf} - \nu_{y\to x})Y, \tag{24}$$

where the four rates on the right hand side correspond to the four processes that can change the number of mutant patches:

- Colonization of empty patches by mutant cells, which increases the number of mutant patches with the per-patch rate

$$\nu_{col} = \mu w_y^{(Y)} \left(1 - \frac{X_{eq} + Z_{eq}}{\mathcal{N}}\right) P^y_{col};$$

[1] Note that this step significantly simplifies all the expressions, but it is not necessary for the method to work. If we keep contributions $Z_x w_x^{(Z_x)}, Z_y w_y^{(Z_y)}$, then the equilibrium value Z_{eq} can be obtained from a quadratic, rather than linear, equation, and the system for mutants will contain linear ODEs for $\dot{Y}$ and $\dot{Z}_y$, where the latter equation can be solved in quasi-equilibrium, reducing the system to a single equation for $\dot{Y}$.

- Conversion of wild-type patches to mutant patches, which also increases the number of mutant patches, with the per-patch rate

$$\nu_{x\to y} = \mu w_y^{(Y)} \frac{X_{eq}}{\mathcal{N}} P_{x\to y};$$

- Infection of mutant patches, which leads to their subsequent extinction (and decreases their number) at the per-patch rate

$$\nu_{inf} = \mu Z_{eq} w_z^{(Z)} \frac{1}{\mathcal{N}} P_{inf}^{y};$$

- Conversion of mutant patches to wild-type patches, which decreases the number of mutant patches with the per-patch rate

$$\nu_{y\to x} = \mu X_{eq} w_x^{(X)} \frac{1}{\mathcal{N}} P_{y\to x}.$$

To better understand the role of these processes in patch selection, we can envisage a corresponding coarse-grained stochastic process, where the number of mutant patches is denoted as $j \in [0, N]$, and during an infinitesimal time, Δt, the following processes take place:

- The number of mutant patches can increase by one with probability $P(j \to j+1) = j(\nu_{col} + \nu_{x\to y})\Delta t$;
- The number of mutant patches can decrease by one with probability $P(j \to j-1) = j(\nu_{inf} + \nu_{y\to x})\Delta t$;
- The number of mutant patches can remain constant with probability $P(j \to j) = 1 - (P(j \to j+1) + P(j \to j-1))$.

Denote by ρ_j the probability that starting from j mutant patches, the system reaches the state where all patches are mutant ($j = N$). We have $\rho_0 = 0$, $\rho_N = 1$, and

$$\rho_j = P(j \to j+1)\rho_{j+1} + P(j \to j-1)\rho_{j-1} + \rho_j(1 - (P(j \to j+1) + P(j \to j-1))), \quad 0 < j < N,$$

which is equivalent to

$$\rho_{j+1}(\nu_{col} + \nu_{x\to y}) + \rho_{j-1}(\nu_{inf} + \nu_{y\to x}) - \rho_j(\nu_{col} + \nu_{inf} + \nu_{x\to y} + \nu_{y\to x}) = 0.$$

The probability of mutant fixation can be obtained by the usual methods (see e.g. [3]) and is given by

$$\rho_i = \frac{1 - 1/(1+s_p)^i}{1 - 1/(1+s_p)^N},$$

where $N \approx X_{eq}$ and the quantity s_p can be interpreted as the effective patch selection coefficient. It is given by

$$s_p = \frac{\nu_{col} + \nu_{x \to y}}{\nu_{inf} + \nu_{y \to x}} - 1. \tag{25}$$

This expression for the effective patch selection coefficient can be rewritten in a more intuitive way:

$$s_p = \frac{R_{ext-rec} s_p^{ext-rec} + R_{conv} s_p^{conv}}{R_{ext-rec} + R_{conv}}, \tag{26}$$

where the rates of the two processes are

$$R_{conv} = \frac{X_{eq}}{\mathcal{N}} \mu w_x^{(X)} P_{y \to x} = \frac{w_x^{(X)} P_{y \to x}}{w_z^{(Z)} P_{inf}^x T_{ext}}, \tag{27}$$

$$R_{ext-rec} = \frac{Z_{eq}}{\mathcal{N}} \mu w_z^{(Z)} P_{inf}^y = \frac{P_{inf}^y w_x^{(X)} P_{col}^x (\mu w_z^{(Z)} P_{inf}^x T_{ext} - 1)}{P_{inf}^x T_{ext} (w_x^{(X)} P_{col}^x + w_z^{(Z)} P_{inf}^x)}, \tag{28}$$

and the associated effective patch selection coefficients are

$$s_p^{conv} = \frac{w_y^{(Y)}}{w_x^{(X)}} \frac{P_{x \to y}}{P_{y \to x}} - 1, \quad s_p^{ext-rec} = \frac{w_y^{(Y)}}{w_x^{(X)}} \frac{P_{col}^y}{P_{col}^x} \frac{P_{inf}^x}{P_{inf}^y} - 1. \tag{29}$$

Note that both expressions (27) and (28) are "out" rates, as they stand for the conversion out of Y and extinction of Y. In the case where the quantities in (29) are small compared to 1, they can be replaced by the "in" rates, and the difference will be small in s_p^{conv} and $s_p^{ext-rec}$.

The regime where the extinction-recolonization process is dominant is characterized by

$$Z_{eq} w_z^{(Z)} P_{inf}^y \gg X_{eq} w_x^{(X)} P_{y \to x},$$

or, denoting by N_{inf} and N_{uninf} the total expected number of infected and uninfected cells at the mutant-free equilibrium, we have the condition

$$N_{inf} P^{y}_{inf} \gg N_{uninf} P_{y \to x}.$$

If the process of extinction-recolonization is predominant, then the probability of mutant fixation, starting from a single fully mutant deme, is given by

$$\rho_{patch} = \frac{1 - 1/(1 + s_e^{ext-rec})}{1 - 1/(1 + s_e^{ext-rec})^{X_{eq}}}. \tag{30}$$

2.4 Comparison with simulations

If the process of conversion is dominant, then we expect the probability of mutant fixation to be similar to that of the Moran process. On the other hand, if the extinction-recolonization process is predominant compared to the conversion process, we expect selection to be significantly weaker and the probability of fixation much larger (smaller) for disadvantageous (advantageous) mutants.

To test this theory, it is instructive to use the parameter regime where the process of extinction-recolonization process is predominant. This is observed when the mutant and wild type cells are typically separated, that is, they do not co-occur in the same demes. Such separation happens for example if many demes are unoccupied (allowing for recolonization by a single type), and when demes' life-span is relatively short (due to intense infection), which precludes accumulation of cells of the different types due to migration.

While spatial separation of mutants and wild-types is typical in simulation with spatially restricted migration, it is more difficult to achieve under non-spatial migration. To increase the degree of separation of mutants and wild types (and thus to amplify the contribution of extinction-recolonization process in the absence of space), we assumed that infected cells migrated at a faster rate than uninfected cells. As a result, demes' lifespans became shorter (due to intense infection) and conversion did not play a significant role.

Supplementary Figure 4 shows a typical result of a stochastic simulation of a non-spatial system where mutant and wild-type cells are largely separated (for a detailed description of the stochastic algorithms used here, see Section 1.1). Mutant individuals were assumed to have a 2% advantage in the division rate. In a simulation run where mutant individuals expand from

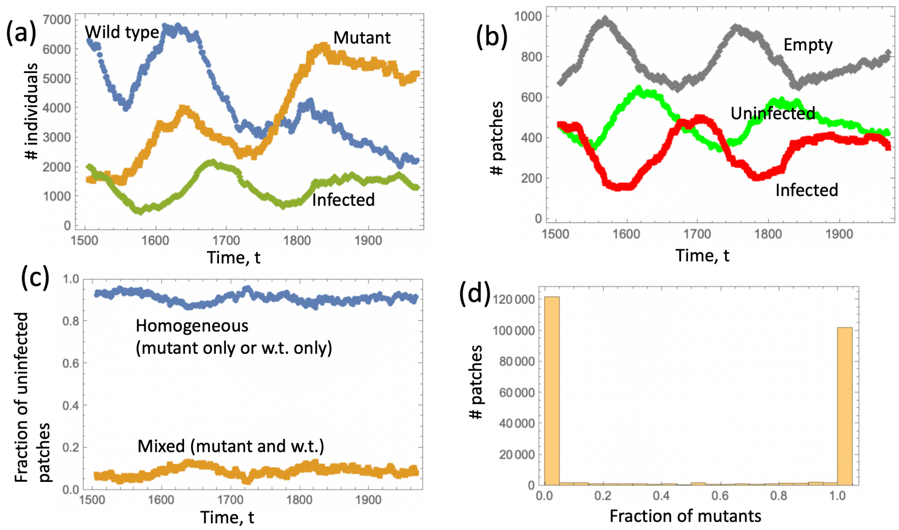

Supplementary Figure 4: Dynamics of a patch system with non-spatial migration. (a) The total number of uninfected wild type (blue) and mutant (orange) individuals, as well as infected (green) individuals, as a function of time. (b) The number of uninfected (green), infected (red), and empty (gray) patches, as a function of time. (c) The fraction of uninfected patches (that is, patches that only contain either mutant or wild type cells), blue, and mixed (both mutant and wild type cells, orange) patches, as a function of time. (d) A histogram showing the number of uninfected nonempty patches characterized by different fractions of mutant cells – a congregate over all 500 time-steps. The parameters are $r_x = 0.7, r_y = 1.02r_x, d_x = d_y = 0.6$, $K = 200, a = 0.1, \beta = 0.04, N = 1600$. The migration rate is $\mu = 0.01$ for uninfected individuals and twice that amount for infected individuals.

low numbers to reach a majority, we recorded the state of the patches once the fraction of mutants reached 20%. Panel (a) shows a time-series for the total (over all patches) numbers of uninfected wild type, uninfected mutants, and infected individuals, to show that the overall number of mutants varies from low to high numbers. Panel (b) shows three types of patches: uninfected (that is, patches that only contain uninfected individuals, regardless of their type), infected (that is, patches that contain at least one infected individual), and empty. In particular, we observe that the number of empty patches is relatively high (the total number of patches is 1600). Panel (c) plots time-series that characterize the degree of mixing of wild type and mutant individuals: for all uninfected nonempty patches, we plot the fraction of patches that either contain mutant or wild type cells (the homogeneous

patches, blue); similarly, in orange, we plot the fraction of mixed patches (that is uninfected patches that contain at least one wild-type and at least one mutant individual). We observe that the fraction of mixed patches stays low as time goes by. Finally, panel (d) shows a histogram of mutant fraction in the uninfected patches. It represents the mutant fractions collected in the course of the simulation, sampled 500 times (after every 10,000 stochastic updates, approximately 1 time unit) during the time-interval depicted in panels (a-c). We can see again that the majority of patches are either 100% mutant or 100% wild-type.

Note that most simulations that start with a low number of mutants will result in mutant extinction without ever seeing a significant number of mutants; for such runs, it is not surprising that most of the patches are homogeneous (and contain wild-type cells). The simulation shown in Supplementary Figure 4 was chosen such that mutants do rise to a significant percentage. It demonstrates that even in such cases, the two types of individuals remain mostly separated (that is, rarely inhabit the same patch).

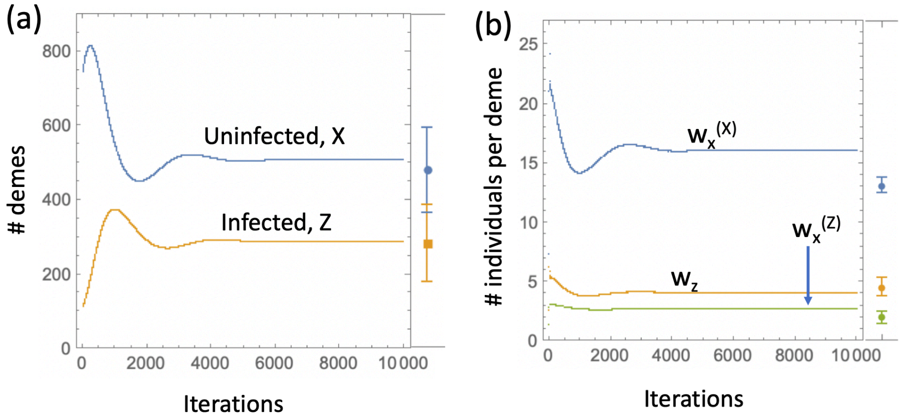

Supplementary Figure 5: The coarse-grained approach applied to the system in Supplementary Figure 4. Iterations of steps (0-6) of section 1.2 are shown for the deme numbers (a) and population sizes (b). The means and standard deviations obtained by stochastic simulations are shown on the right side of each graph. Parameters are as in Supplementary Figure 4.

For this system, we demonstrate that the method described above produces a very good prediction of the mutant fixation probability. Supplementary Figure 5 shows the result of iterations of system (15-16), to obtain

an approximation of the deme numbers and population sizes. The means and standard deviations obtained by stochastic simulations are shown on the right side of each graph.

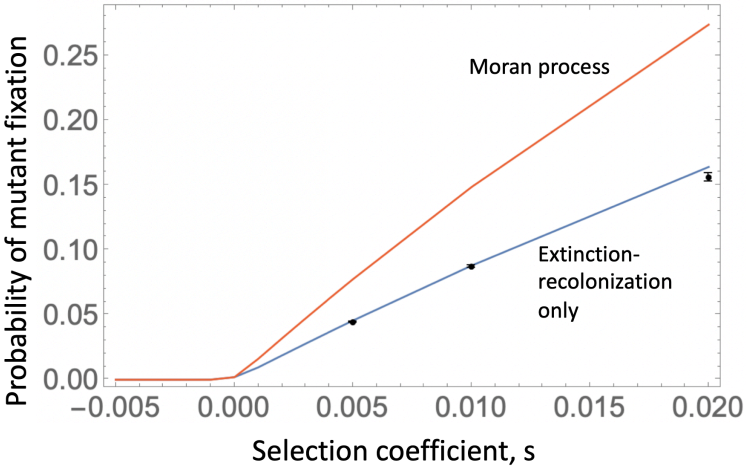

Supplementary Figure 6: Mutant fixation probability as a function of mutant selection coefficient, s. The blue line is the coarse-grained prediction, equation (30). The red line is the Moran prediction, equation (31). The points with small vertical bars represent the mean and standard deviation of the probability of fixation determined by stochastic simulations for three different values of s. The rest of the parameters are as in Supplementary Figure 4.

The probability of mutant fixation, starting with a single fully-mutant deme, is given by equation (30). Supplementary Figure 6 compares this calculation (blue line) with the result for the usual Moran prediction,

$$\rho_{Moran} = \frac{1 - 1/(1+s)^{w_x^{(X)}}}{1 - 1/(1+s)^{w_x^{(X)} X_{eq}}}, \tag{31}$$

which is shown by the red line. Numerical simulations of the stochastic system were performed to estimate the probability of mutant fixation; the results are shown by points with small standard deviations that correspond to three different values of the mutant selection coefficient s. In these simulations, first, a patch system in the absence of mutants was allowed to reach a quasi-equilibrium. Then, at a time when the number of uninfected cells was given by N_u (the numerically determined mean number at quasi-equilibrium), a

patch that contained at least one uninfected cell was selected randomly, and all uninfected cells were replaced by mutant uninfected cells. The simulation was stopped when either the mutants reached fixation or were extinct. This process was repeated $15,480$ times, for each value of s, yielding the fraction of the runs that resulted in mutant fixation.

We observe that the prediction of the coarse-grained approach is very similar to the observed probability of mutant fixation, and that the Moran prediction corresponds to a much higher fixation probability for advantageous mutants.

References

[1] Rajesh Ramaswamy, Nélido González-Segredo, and Ivo F Sbalzarini. A new class of highly efficient exact stochastic simulation algorithms for chemical reaction networks. *The Journal of chemical physics*, 130(24), 2009.

[2] Todd L Parsons and Christopher Quince. Fixation in haploid populations exhibiting density dependence I: the non-neutral case. *Theoretical population biology*, 72(1):121–135, 2007.

[3] Samuel Karlin and Howard E Taylor. *A second course in stochastic processes*. Elsevier, 1981.
