# Supplementary material for: Mutant fixation in the presence of a natural enemy
Source: Nat Commun. 2023 Oct 20;14:6642. doi: 10.1038/s41467-023-41787-5 (PMC10589345; doi:10.1038/s41467-023-41787-5)
Supplement: Supplementary file 3 — Description of additional supplementary files [file 41467_2023_41787_MOESM3_ESM.pdf]

Description of Additional Supplementary Files

Supplementary Code 1: This is the code that generates numerical results for the agent-based model described in the paper.

Supplementary Code 2: This is the code that generates numerical results for the non-spatial deme model described in the paper.

Supplementary Code 3: This is the code that generates numerical results for the spatial deme model described in the paper.
